# Sjogren’s Syndrome Complicated With Aldosterone-Producing Adenoma: A Case Report

**DOI:** 10.7759/cureus.45793

**Published:** 2023-09-22

**Authors:** Xiaohuan Chen, Bo Lou, Yulan Hu, Huanhuan Ma, Jiacheng Shi, Pengfei Shan

**Affiliations:** 1 Department of Endocrinology and Rheumatology, The First People's Hospital of Linping District, Hangzhou, CHN; 2 Department of Nephrology, Haining People's Hospital, Jiaxing, CHN; 3 Department of Endocrinology and Metabolism, The Second Affiliated Hospital of ZheJiang University School of Medicine, Hangzhou, CHN

**Keywords:** sjogren’s syndrome, inflammatory rheumatoid disease, aldosteronism, other causes of hypokalemia, aldosterone-producing adenoma

## Abstract

Hypokalemia may be present in some patients with Sjogren's syndrome. When a patient with Sjogren's syndrome presents with hypokalemia, we would first consider it to be a result of the renal involvement of Sjogren's syndrome. However, in this case report, we present a young woman with Sjogren's syndrome who presented with hypokalemia that was not caused by renal tubular acidosis but by the presence of a coexisting aldosterone-producing adenoma. Cases of Sjogren's syndrome coexisting with aldosterone-producing adenoma are extremely rare. This finding underscores the need for more careful differential diagnosis in patients with Sjogren's syndrome who also have hypokalemia.

## Introduction

Sjogren's syndrome is a chronic autoimmune disease characterized by focal lymphocyte infiltration and invasion of exocrine glands, especially salivary and lacrimal glands [1,2]. Dry keratoconjunctivitis and xerostomia are the primary clinical symptoms of Sjogren's syndrome. The kidney, neurological system, digestive system, blood system, skin, skeletal muscle, respiratory system, etc. may also be affected by Sjogren's syndrome. Patients often exhibit hypergammaglobulinemia and autoantibodies in serum tests [1-4]. Case reports of Sjogren's syndrome combined with aldosterone-producing adenoma were extremely rare. We report a case of a young woman with Sjogren's syndrome who presented with hypokalemia and hypertension, thereby identifying a concomitant case of aldosterone-producing adenoma.

## Case presentation

**Figure 1:**
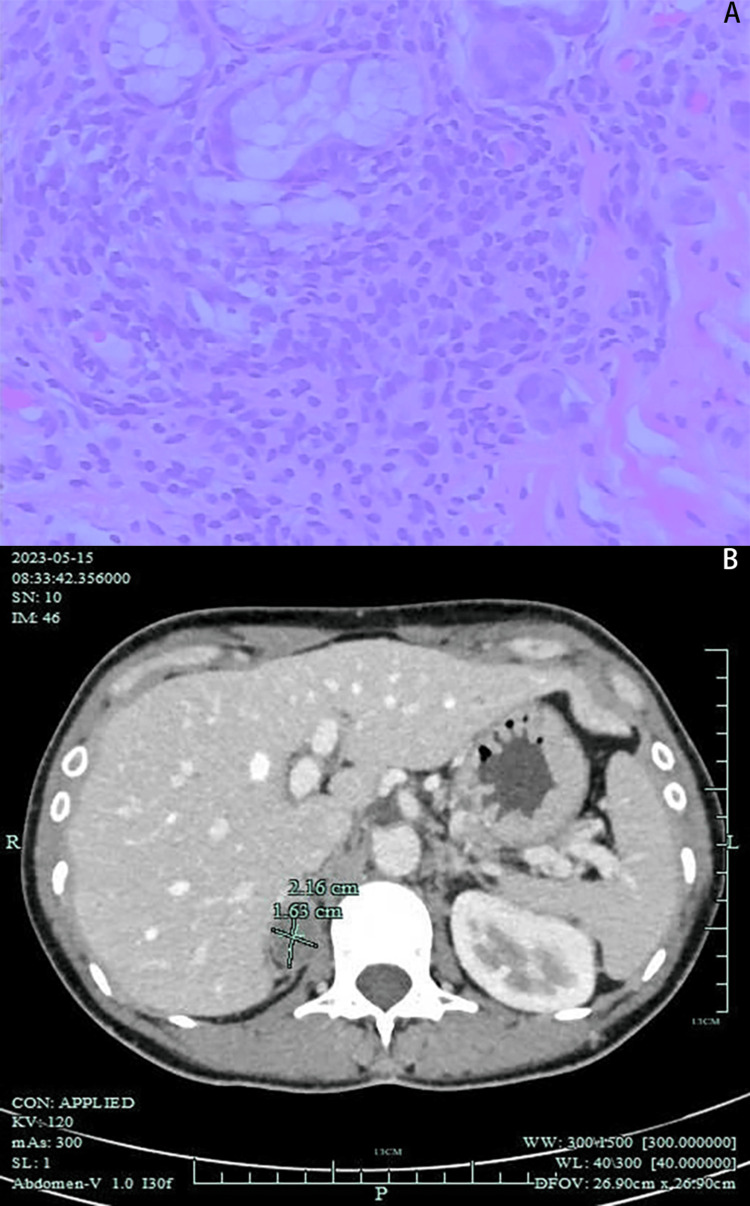
Pathology of the labial gland biopsy and contrast-enhanced CT of the adrenal gland A: Focal lymph follicle infiltration revealed in a labial salivary gland biopsy (hematoxylin and eosin stain, 400× magnification) in a sample taken from a 30-year-old Chinese woman presenting with Sjogren’s syndrome complicated with aldosterone-producing adenoma. B: Contrast-enhanced CT of the adrenal gland showed a right adrenal nodule with a size of about 22×16 mm, which was slightly enhanced, and the shape and contour of the left adrenal gland were normal without obvious abnormal density changes. There were no obvious enlarged lymph nodes in the retroperitoneum.

A 30-year-old lady who had experienced dry mouth and dry eyes for more than four years was admitted to the First People's Hospital of Linping District (Hangzhou, China) in May 2023. She did not have a definite cause for her dry mouth and eyes four years ago. She has difficulty with eating dry foods. When her hands become cold, the tips of the fingers turn white. Red rashes sporadically emerge on the face after exposure to the sun. She experiences headaches, dizziness, fatigue, and chest tightness when she is tired. The patient's antinuclear antibody (ANA) titer was 1:320 (reference range: <1:80) in the outpatient examination, and the anti-SSA antibody, anti-Ro antibody, and anti-SSB antibody were positive; hence, she was admitted to the department of Endocrinology and Rheumatology for further examination. When the patient was admitted, her blood pressure was 149/105 mmHg, other vital signs were all normal, and a physical examination turned up nothing unusual. Initial lab investigations revealed serum potassium was 2.4 mmol/L, complement 3 was 0.61 g/L. Routine blood tests, liver function, renal function, blood lipids, high-sensitivity C-reactive protein, thyroid function, erythrocyte sedimentation rate (ESR), tumor markers, hepatitis B, hepatitis C, syphilis, and human immunodeficiency virus (HIV) antibodies were normal. Other rheumatic immune-related indicators were also normal, including human leucocyte antigen-B27 (HLA-B27), cytoplasmic antineutrophil cytoplasmic antibodies (cANCA), perinuclear antineutrophil cytoplasmic antibodies (pANCA), proteinase 3 (PR3), myeloperoxidase (MPO), phospholipid antibodies, rheumatoid factor, and immunoglobulin. A urine routine test showed a urine pH of 7.0. The pathology of the labial gland biopsy revealed two foci of lymphocyte infiltration (more than 50 cells in each foci) and several glandular lobes in the tissue under examination (Figure 1A). After consulting with an ophthalmologist, dry eye was identified, and the Schirmer test came back positive. A thin-slice computerized tomography (CT) scan of the chest showed small nodules in both lungs, and proliferative calcification was considered. There was a small cystic lesion in the right liver and a hypodense lesion in the right adrenal region. According to the 2016 ACR/EULAR diagnostic criteria for Sjogren's syndrome [5], the patient complained of dry mouth and dry eyes. Moreover, the pathology of the labial gland showed that there were two lymphocyte lesions, a positive anti-SSA antibody and anti-Ro antibody and a positive Schirmer test, so the diagnosis of Sjogren's syndrome was clear. But what was the cause of the patient's hypokalemia, due to renal tubular acidosis caused by Sjogren's syndrome or adrenal gland problem?

After symptomatic treatment with potassium supplementation, the serum potassium level was still low, and the blood pressure was always high during hospitalization. Further evaluation was performed on this patient. The blood gas analysis showed that the blood pH was 7.47, the actual bicarbonate ion was 33.4 mmol/L, the actual remaining alkali was 9 mmol/L, the potassium ion was 2.6 mmol/L, and the total carbon dioxide was 34.8 mmol/L. The 24-hour urine potassium was 52 mmol/L. The orthostatic aldosterone was 22.298 ng/dL, the renin activity was 0.65 ng/mL/h, and the plasma aldosterone to renin activity ratio (ARR) was 34.3(ng/dL)/(ng/mL/h), which was considered to be primary aldosteronism. The results of the saline suppression test showed that the level of aldosterone was 38.695 ng/dL after infusion of 2000 mL saline. The results of the captopril inhibition test showed that aldosterone was 30.251 ng/dL two hours after medication. Adrenal contrast-enhanced CT showed a right adrenal nodule, and an adenoma was considered (Figure 1B). Blood gas analysis showed metabolic alkalosis, and aldosterone was not suppressed in the normal saline suppression test and captopril suppression test. Thus, we considered that the patient was diagnosed with Sjogren's syndrome with aldosterone-producing adenoma. The patient's persistent hypokalemia and elevated blood pressure were not due to renal tubular acidosis but rather to aldosteronism that resulted from an aldosterone-producing adenoma. After consultation with the Urology department, the patient was sent to the Urology department for right adrenal lesion resection. Postoperative pathology showed that the right adrenocortical adenoma (size 4 × 2.5 × 1.5 cm), and some cells grew actively. The patient's serum potassium returned to normal at discharge, and she mainly took immunomodulatory drugs for Sjogren's syndrome, including hydroxychloroquine sulfate and total glucosides of paeony capsules. One month after surgery, the patient's serum potassium was 4.00 mmol/L and blood pressure was 124/77 mmHg. Three months after surgery, the patient's upright aldosterone was 3.508 ng/dL, renin activity was 0.2 ng/mL/h, ARR was 17.54 (ng/dL)/(ng/mL/h), and blood pressure was 110/71 mmHg. The patient felt dry mouth and dry eyes better than before, and there was no obvious fatigue, chest tightness, or other discomfort. Presently, the patient remains under follow-up observation.

## Discussion

Patients with Sjogren's syndrome often present with hypokalemia, which is associated with renal damage in Sjogren's syndrome. The kidney is one of the most important target organs of Sjogren's syndrome, which is mainly characterized by renal tubulointerstitial damage [1,2]. The clinical manifestations are hypokalemia and renal tubular acidosis. In patients with Sjogren's syndrome complicated with renal damage, distal renal tubules can be involved, resulting in decreased hydrogen secretion, increased hydrogen ions in blood, and decreased blood pH value. When acidosis occurs, the potassium ions excreted with urine increase, leading to a decrease of blood potassium and hypokalemic paralysis. Patients with Sjogren's syndrome may have limb weakness, and in severe cases, dyspnea and other symptoms may occur. Due to the insidious onset of renal damage caused by Sjogren's syndrome, the diagnosis sometimes relies on renal tubular function examination or renal biopsy [6]. Therefore, renal damage caused by Sjogren's syndrome is easily misdiagnosed in the early stage. In clinical work, when hypokalemia occurs in patients with Sjogren's syndrome, we often think of Sjogren's syndrome involving the kidney first and ignore other factors. The causes of hypokalemia are complex, including insufficient intake, excessive excretion, and abnormal distribution. Hypokalemia can cause symptoms such as arrhythmias, fatigue, muscle soreness, constipation, ileus, and even paralysis [7]. Therefore, for patients with hypokalemia, we also need to identify the cause of hypokalemia in the process of potassium supplement treatment.

Primary hyperaldosteronism refers to the spontaneous secretion of aldosterone by the adrenal cortex, which leads to sodium retention and potassium excretion in the body, increased blood volume, and inhibition of the activity of the renin-angiotensin system. Its main clinical manifestations are hypertension and hypokalemia. Primary aldosteronism can be divided into six types according to different causes, including aldosterone-producing adenoma, idiopathic hyperaldosteronism, primary or unilateral hyperplasia, familial hyperaldosteronism, aldosterone-producing adrenocortical carcinoma, and ectopic aldosterone-producing adenoma [8]. As the most commonly used screening index for primary aldosteronism, ARR has been widely used in clinical practice. Confirmatory tests for primary aldosterone mainly include saline and captopril tests. In this case, the patient started with a dry mouth and dry eyes. According to the laboratory results and the pathological result of the labial gland, the diagnosis of Sjogren's syndrome is clear, but what is the cause of the patient's persistent hypokalemia and hypertension? She had a normal diet, and underconsumption was not considered to be the cause. She does not have gastrointestinal symptoms such as nausea, vomiting, and diarrhea, and excessive gastrointestinal discharge was not considered to be the cause. Based on the results of the urine routine and blood gas analysis, hypokalemia caused by renal tubular acidosis was excluded. According to the 24-hour urinary electrolytes, the cause of hypokalemia was considered to be excessive potassium excretion. After completing the aldosterone and renin tests, we found that this patient had a high aldosterone level, low renin level, and high ARR. In view of the patient's chest CT finding a right adrenal incidentaloma, we proceeded to complete the normal saline test, captopril test, and enhanced CT of the adrenal gland, and we finally confirmed primary aldosteronism. Then, the pathological results were obtained by surgical resection of the affected adrenal gland, and the final diagnosis was aldosterone-producing adenoma. The patient's hypokalemia and hypertension were corrected immediately after the operation.

Currently, there is a lack of research on the correlation between Sjogren's syndrome and aldosterone-producing adenoma. The occurrence of both is extremely rare. Therefore, in this patient, we consider a dualistic interpretation that would suggest that this patient has both Sjogren's syndrome and aldosterone-producing adenoma. Her dry mouth and dry eyes were caused by Sjogren's syndrome, and her hypokalemia and hypertension were caused by aldosterone-producing adenomas.

## Conclusions

In conclusion, we report a case of Sjogren's syndrome complicated by aldosterone-producing adenoma. Through retrospective analysis of the diagnosis and treatment of this patient and a review of the relevant literature, we concluded that hypokalemia in patients with Sjogren's syndrome should not be considered only because of renal involvement. In clinical work, we need to further evaluate whether there are other causes of hypokalemia in combination with the actual situation of patients with Sjogren's syndrome. Early diagnosis and timely treatment may allow for better control and prevent the progression of the disease.
